# TGF‐β/Smad and Wnt/β‐catenin signaling pathways are involved in renal fibrosis and its therapies

**DOI:** 10.1002/ctm2.127

**Published:** 2020-07-07

**Authors:** Jialiang Ying, Xin Han, Xia'nan Sang, Gang Cao

**Affiliations:** ^1^ School of Pharmacy Zhejiang Chinese Medical University Hangzhou China

Dear Editor,

The chronic kidney disease normally results in renal fibrosis, and accompanied by glomerulosclerosis and tubulointerstitial fibrosis. Chronic kidney disease is one of the biggest challenges in nephrology, as it will lead patients inevitably to reach end‐stage of renal disease, and experience replacement therapies, such as hematodialysis and renal transplantation. Renal fibrosis can be meditated by a variety of pathways, such as renin‐angiotensin system (RAS), Notch, Hedgehog, as well as signals and signaling channel of TGF‐β/Smad and Wnt/β‐catenin. Meanwhile, many small‐molecule inhibitors can treat this disease by means of suppressing epithelial‐mesenchymal transition (EMT). Natural products have the potential to intervening fibrosis in both one organ and multiple organs and thus provide a new therapeutic strategy for antifibrosis (Table 1). For example, new tetracyclic triterpenoids (poricoic acids) from the surface layer of *Poria cocos* can restrain the renin‐angiotensin system, and so does the activation of signals and signaling pathways of TGF‐β1/Smad and Wnt/β‐catenin.^1^, ^2^ Relevant therapeutic mechanism is shown in Figure 1.

**TABLE 1:** Natural product related to renal fibrosis

Compounds	Source	Signaling pathways
Schisandrin B	*Schisandra chinensis*	TGF‐β
Poricoic acid ZA	*Poria cocos*	RAS, TGF‐β/Smad
Poricoic acid ZC, Poricoic acid ZD, Poricoic acid ZE	*Poria cocos*	TGF‐β/Smad, Wnt/β‐catenin
Poricoic acid ZF, Poricoic acid ZG, Poricoic acid ZH	*Poria cocos*	RAS, TGF‐β/Smad3
25‐O‐methylalisol F	*Alisma orientale*	RAS, TGF‐β/Smad3, Wnt/β‐catenin
Alisol B 23‐acetate	*Alisma orientale*	Wnt/β‐catenin
Ergone	*Polyporus umbellatus*	TGF‐β/Smad, Wnt/β‐catenin
Pterostilbene	Blueberries	IκB/NF‐κB/miR‐488, TGF‐β1
Berberine	*Berberis* Genus	IκB/NF‐κB, TGF‐β/Smad, JAK/STAT
Salvianolic acid A	*Salvia miltiorrhiza* Bunge	TGF‐β, p53
Epigallocatechin‐3‐gallate	Green tea	TGF‐β/Smad2, Keap1/Nrf2
Wogonin	*Scutellaria baicalensis* Georgi	TGF‐β/Smad3
Oxymatrine	*Radix sophorae flavescentis* Genus	TGF‐β/Smad3
Rutin	The wide variety of plants	TGF‐β/Smad3
Hesperetin	*Stilbella fimetaria*	Hedgehog, TGF‐β/Smad, IκB/NF‐κB
Acetyl‐11‐keto‐β‐boswellic acid	*Boswellia serrate*	TGF‐β/Smad
Betulinic acid	*Betula alba*	TGF‐β/Smad
Hydroxysafflor Yellow A	Safflower	TGF‐β/Smad

**FIGURE 1:**
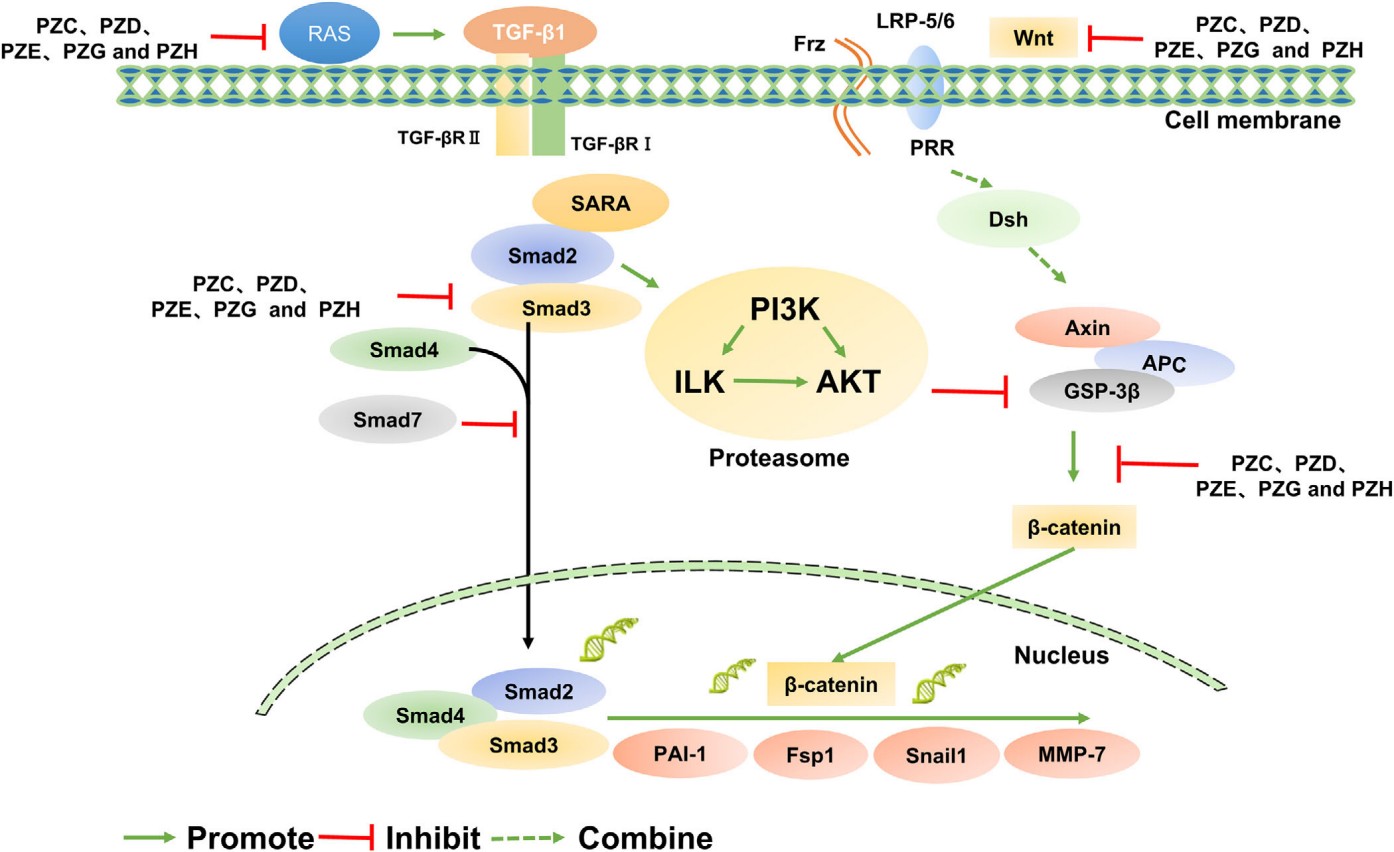
Therapeutic mechanism related to natural products. The mechanism of action of poricoic acids, poricoic acid ZG (PZG), poricoic acid ZH (PZH), poricoic acid ZC (PZC), poricoic acid ZD (PZD), and poricoic acid ZE (PZE), which are novel RAS inhibitors. The figure shows the interaction between TGF‐β/Smad signaling pathway and Wnt/β‐catenin signaling pathway. Among them, natural products selectively inhibit the phosphorylation of Smad3 induced by TGF‐β1 by inhibiting RAS and blocking the interaction between TGF‐βRI and Smad3. At the same time, it can eventually inhibit renal fibrosis by reducing the expression levels of Wnt and β‐catenin proteins

Modern clinical pharmacological studies have shown that traditional Chinese medicines have extensive bioactivities and play a wide spectrum of important roles in regulating immune function and exert their anticancer, antiinflammatory, and antifibrotic effects. *ShenKang* injection is a traditional Chinese medicine preparation prepared from *Rhubarb*, *Astragalus*, *Radix Salviae Miltiorrhizae*, and *Carthami Flos*. It ameliorates glomerulosclerosis and tubulointerstitial fibrosis including the inhibition of collagen I, α‐smooth muscle actin p‐smad3, and fibronectin through inhibiting TGF‐β/Smad3 signaling pathway.^3^ *You‐gui* pill treatment can effectively protect kidney function and attenuate tubulointerstitial fibrosis, which are related to TGF‐β expression and its signaling molecules, Smad2, or Smad3.^4^ The combination of poricoic acid A and melatonin interacts to inhibit the progression of renal fibrosis by regulating signaling molecules, such as Smad‐3 and β‐catenin, during renal fibrosis.^5^ *Wulingsan* that is a classical Chinese compound prescription and contains *Alisma orientalis*, *Polyporus umbellatus*, *Poria cocos*, and *Atractylodes macrocephala* commonly used to treat chronic kidney disease (CKD) clinically.^6^

In conclusion, renal fibrosis is a dynamic, progressive, and irreversible process. It is a common pathway for the progression of various CKD. At present, the mechanism associated with signaling pathways in renal fibrosis remains enigmatic. Wnt/β‐catenin and TGF‐β/Smad signalings are two essential pathways in renal fibrosis. They mediate and participate in the process of renal fibrosis. Although they have their own mechanism systems, they promote and complement in renal fibrosis. However, studies on the synergetic mechanism of two signaling pathways should be further explored. At present, many drugs in clinic that are used to treat renal fibrosis exert their therapeutic effects through targeting RAS, Wnt/β‐catenin, p‐Smad3, and their interaction. However, these drugs can only alleviate or inhibit the progression of renal fibrosis and cannot direct target completely renal fibrosis. In the future, additional fibrosis‐specific regulatory molecules related to the pivotal factors in signaling pathways of Wnt/β‐catenin or TGF‐β/Smad should be studied in accordance with the roles of these different approaches in renal fibrosis’ progression. Advantages of emerging natural active molecules, new drugs, or traditional Chinese medicines for effective treatment of renal fibrosis still needs to be studied for a long time.

## CONFLICT OF INTEREST

The authors declare that there is no conflict of interest.
